# The pathophysiology of Sumatriptan induced non-arteritic anterior ischemic optic neuropathy

**DOI:** 10.22336/rjo.2022.62

**Published:** 2022

**Authors:** Dana Dăscălescu, Cătălina Corbu, Livia Șram, Valeria Coviltir, Mihaela Constantin, Miruna Burcel, Maria Marinescu, Andrei Comber, Vasile Potop

**Affiliations:** * Department of Ophthalmology, “Carol Davila” University of Medicine and Pharmacy Bucharest, Romania; ** Department of Ophthalmology, Clinical Hospital for Ophthalmological Emergencies Bucharest, Romania; *** Department of Ophthalmology, Oftaclinic Bucharest, Romania

**Keywords:** NAAION, Sumatriptan, migraine

## Abstract

**Objective:** To report a case of a non-arteritic anterior ischemic optic neuropathy (NAAION) in a patient treated with Sumatriptan.

**Materials and methods:** NAAION represents a severe affection that frequently determines irreversible visual acuity damage. The exact cause is yet to be identified, but is usually connected to the systemic status of the patient. We presented the case of a 53-year-old female patient who complained of visual acuity loss in her right eye, associated with inferior visual field (VF) damage. Patient history revealed migraine attacks, raised arterial blood pressure (BP), mitral valve insufficiency and dyslipidemia. Systemic treatment included Sumatriptan for migraine attacks and Bisoprolol for arterial hypertension.

**Results:** A complete ophthalmologic examination was performed, including a visual field examination and optic coherence tomography. Interdisciplinary consults, along with inflammatory biomarkers, brain scan and cardiovascular Doppler echography were used to establish the final diagnosis. Considering the patient’s history, systemic medication, clinical picture, paraclinical findings and interdisciplinary check-ups, NAAION was established as a diagnosis.

**Discussion:** NAAION occurs more frequently after the age of 50 years old and may be associated with systemic factors such as nocturnal hypotension, diabetes, atherosclerosis, sleep apnea. In the present case, the association of medically induced nocturnal hypotension and vasoconstriction led to optic nerve ischemia.

**Conclusions:** In a patient with multiple pathology, we must consider the systemic therapy when performing any clinical examination.

**Abbreviations:** AAION = arteritic anterior ischemic optic neuropathy, AION = anterior ischemic optic neuropathy, BCVA = best corrected visual acuity, BP = blood pressure, CS = corticosteroid, IOP = intraocular pressure, LE = left eye, MRI = magnetic resonance imaging, NAAION = non-arteritic anterior ischemic optic neuropathy, OCT = optical coherence tomography, ON = optic nerve, OU = both eyes, RE = right eye

## Introduction

Anterior ischemic optic neuropathy (AION) represents one of the most important causes of unilateral optic nerve (ON) related visual acuity loss in an individual older than 50 years [**1**,**2**]. AION has two forms: arteritic and non-arteritic [**1**]. The exact mechanism is not entirely understood and new causes are still under investigation, but most likely includes decreased perfusion in the short posterior ciliary arteries (SPCA), which are responsible for ON head blood flow [**2**]. Arteritic AION (AAION) is subsequent to giant cell arteritis that determines inflammation on medium vessels (e.g., the temporal artery), leading to decreased perfusion in the eye [**1**,**2**]. Non-arteritic AION (NAAION) is found in patients who associate vascular risk factors including sleep apnea syndrome, diabetes mellitus or arterial hypertension [**1**-**3**].

## Materials and methods

We presented the case of a 53-year-old patient who came to the Emergency Room, complaining of recent, painless visual acuity loss in her right eye (RE), associated with inferior visual field (VF) damage. Personal history included chronic migraines (for about 20 years), mitral valve insufficiency, dyslipidemia, and arterial hypertension. She used Sumatriptan as a therapy for migraine attacks and Bisoprolol as an antihypertensive agent. The patient described an unusual headache and started to use Sumatriptan at night, shortly after Bisoprolol was administered. In the following morning, she woke up with the visual acuity (VA) damage.

## Results

**Fig. 1:**
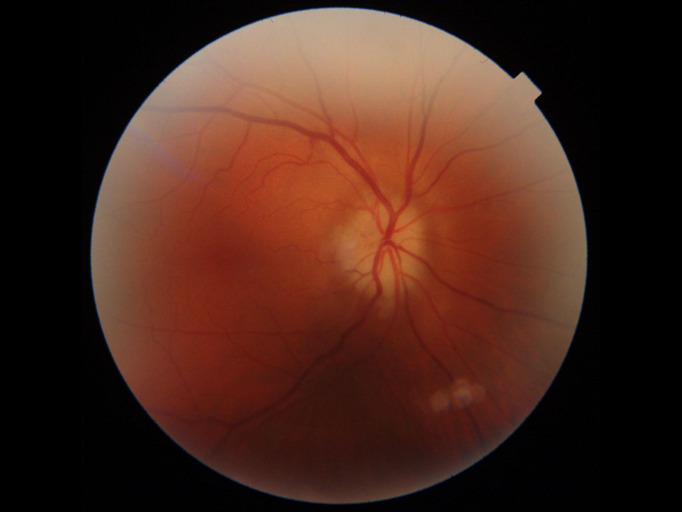
Optic nerve aspect in the RE

Ophthalmologic examination showed a best corrected visual acuity (BCVA) of 0.2 Snellen in the RE and of 1 Snellen in her left eye (LE). Intraocular pressure measurement (IOP) was 15 mmHg in both eyes (OU). Slit lamp examination, pupillary reflexes and eye motility were normal. Fundus evaluation in the RE displayed elevated optic disc, attached retina, generalized arteriolar narrowing and dilated veins (**Fig. 1**), while the fundus evaluation in the LE showed cup/ disc ratio 0.2, attached retina, generalized arteriolar narrowing and dilated veins. 

Visual field assessment detected an inferior altitudinal scotoma in the RE, and normal VF in the LE. Ocular echography objectivated the papillary edema, with no calcifications. Optical Coherence Tomography (OCT) also confirmed these findings. Paraclinical investigations showed a normal inflammatory profile and hypercholesterolemia. Doppler echography was normal. Neurology consultation was normal and cerebral MRI showed some microangiopathic lesions. Cardiology consultation revealed mitral insufficiency, arterial hypertension and dyslipidemia. Differential diagnosis included NAAION, AAION, papillitis, retrobulbar optic neuritis, papillary edema, and optic nerve drusen. Based upon the ophthalmologic examination, interdisciplinary checkups, paraclinical data and Imagistics, the positive diagnosis of NAAION was established.

Considering the risk-benefit ratio of corticosteroid (CS) treatment, regarding a relatively young patient, without diabetes, a stable blood pressure (BP) and no major risk factors, we decided to administer the CS, additional to a platelet aggregation inhibitor, peripheral vasodilator and neurotrophic agent under surveillance, monitoring the patient closely. Short-term evolution was stable, without further visual acuity loss, but also without an improvement.

**Fig. 2:**
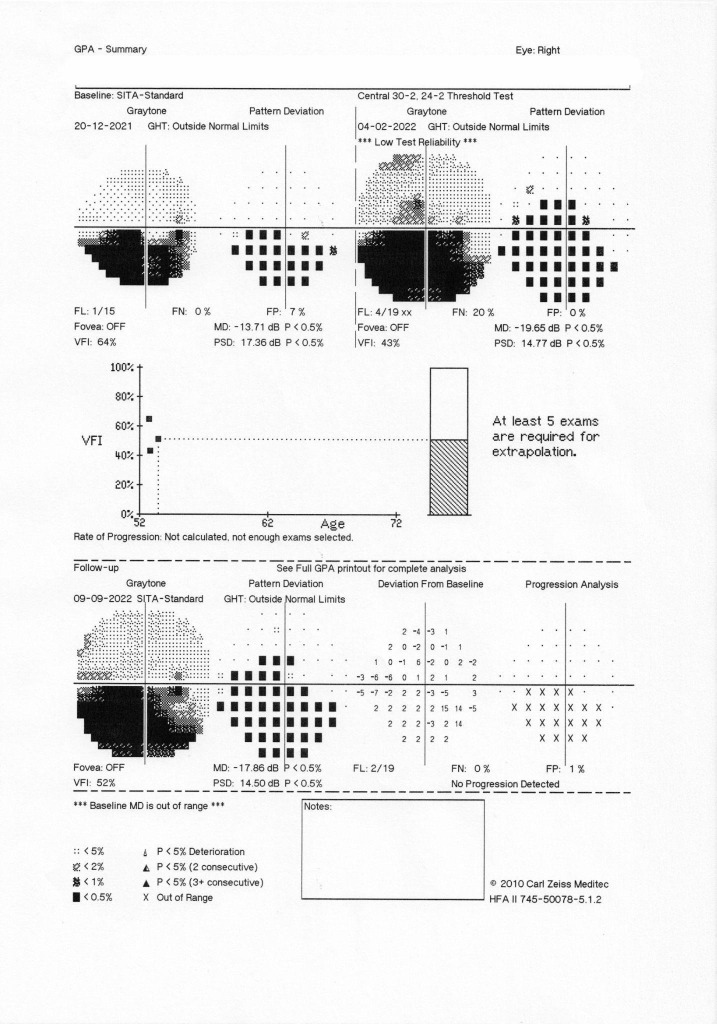
Visual field evolution RE

**Fig. 3:**
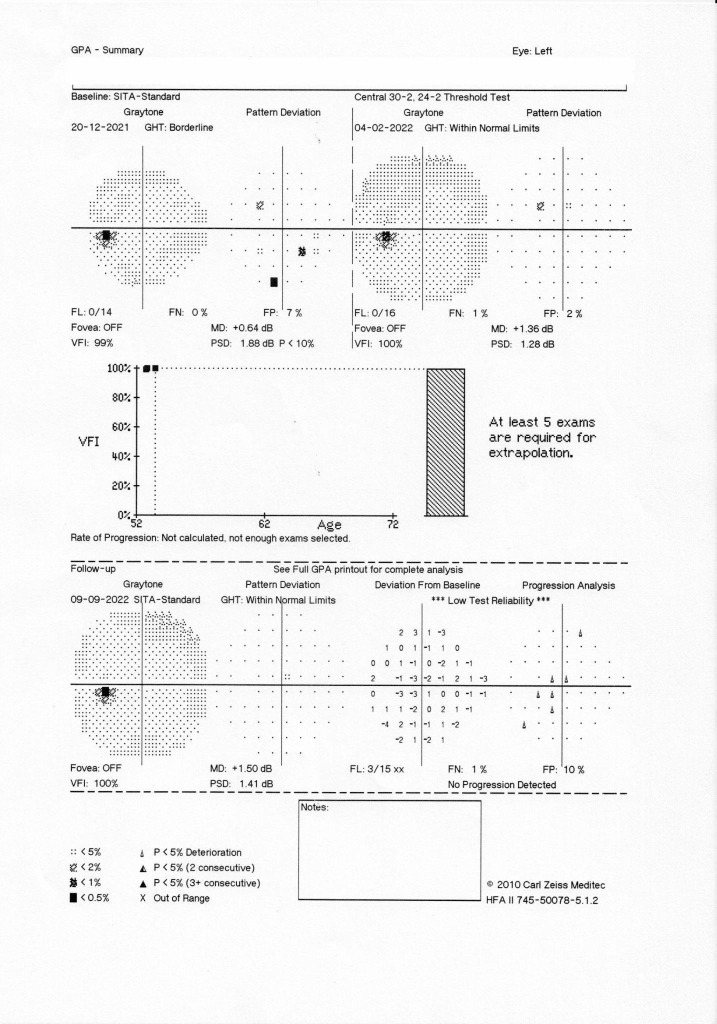
Visual field evolution LE

Since the patient was administered a hypotensive agent in the evening and a vasoconstrictor at night and woke up with NAAION, the possible relationship between these events was discussed. After the interdisciplinary checkups with neurology and cardiology, we decided to interrupt the Sumatriptan treatment and to monitor de arterial BP closely, especially at night. Long-term evolution revealed a pale disc in the RE, while LE was stationary (**Fig. 2**, **3**).

The visual field showed an initial increase in scotoma size, but without further changes after Sumatriptan was stopped.

## Discussion

NAAION occurs more frequently in patients over 50 years old with a structurally crowded disc and known with nocturnal hypotension, diabetes, atherosclerosis, smoking and sleep apnea [**1**,**3**-**5**]. Although the exact cause is rarely identified, many patients associate factors that affect the vascular blood flow in the SPCA that irrigate the anterior ON [**4**,**6**,**7**]. The hemifield VF defect associated to AION can be explained considering that the ON head blood supply involves arterial branches from the circle of Zinn-Haller that arises out of the SPCA [**4**,**8**].

Migraines represent an acute headache of neurovascular cause that may associate nervous system malfunction or an aura [**8**]. Triptans are a class of selective serotonin receptor agonists, binding to the 5-hydroxytryptamine 1B and 1D receptors and relieving the headache by vasoconstriction and central neural inhibition [**8**]. Studies show that Sumatriptan does not prevent migraines, but given subcutaneously, intravenously, or orally soon after the pain starts, is effective in treating an acute attack of migraine. It relieves pain, photophobia, nausea and phonophobia, but may be related to a high risk of adverse effects [**8**,**9**]. 

Nocturnal hypotension can be determined by antihypertensive treatment administered extensively in the evening without a close monitoring of BP values. The vasoconstriction related to a low perfusion rate can determine severe consequences due to ischemia in the small blood vessels. 

Presently, the patient is administered Bisoprolol at the end of the day, followed by Sumatriptan several hours later as relief at the start of a migraine attack. The hypotensive and vasoconstrictor effects led to acute ischemia in the SPCA, which manifested as inferior hemifield defect, due to NAAION, upon waking.

## Conclusion

In front of a patient suffering from Sumatriptan-responsive migraines, we need to consider the systemic associations and medication aiming to minimize the adverse effects. Associating nocturnal hypotension and Sumatriptan administered at night can determine NAAION.

**Conflict of Interest statement**

The authors state no conflict of interest.

**Informed Consent and Human and Animal Rights statement**

Informed consent has been obtained from the individual included in this study.

**Authorization for the use of human subjects**

Ethical approval: The research related to human use complies with all the relevant national regulations, institutional policies, is in accordance with the tenets of the Helsinki Declaration, and has been approved by the review board of the Clinical Hospital for Ophthalmological Emergencies Bucharest, Romania.

**Acknowledgements**

None.

**Sources of Funding**

None.

**Disclosures**

None.

**Contribution**

All authors had equal contribution in the paper.
